# GeomeTRe: accurate calculation of geometrical descriptors of tandem repeat proteins

**DOI:** 10.1093/bioinformatics/btaf395

**Published:** 2025-07-10

**Authors:** Zarifa Osmanli, Elisa Ferrero, Alexander Miguel Monzon, Silvio C E Tosatto, Damiano Piovesan

**Affiliations:** Department of Biomedical Sciences, University of Padova, Padova, 35121, Italy; Department of Biomedical Sciences, University of Padova, Padova, 35121, Italy; Galileian School of Higher Education, University of Padova, Padova, 35132, Italy; Department of Biomedical Sciences, University of Padova, Padova, 35121, Italy; Department of Biomedical Sciences, University of Padova, Padova, 35121, Italy; Institute of Biomembranes, Bioenergetics and Molecular Biotechnologies, National Research Council (CNR-IBIOM), Bari, 70126, Italy; Department of Biomedical Sciences, University of Padova, Padova, 35121, Italy

## Abstract

**Motivation:**

Structured tandem repeat proteins (STRPs) are characterized by preserved structural motifs arranged in a modular way. The structural and functional diversity of STRPs makes them particularly important for studying evolution and novel structure–function relationships, and ultimately for designing new synthetic proteins with specific functions. One crucial aspect of their classification is the estimation of geometrical parameters, which can provide better insight into their properties and the relationship between the spatial arrangement of repeated units and protein function. Calculating geometric descriptors for STRPs is challenging because naturally occurring repeats are not “perfect” and often contain insertions and deletions. Existing tools for predicting structural symmetry work well on simple cases but often fail for most natural proteins.

**Results:**

Here, we present GeomeTRe, an algorithm that calculates geometrical descriptors such as curvature (yaw), twist (roll), and pitch for a protein structure with known repeat unit positions. The algorithm simulates the movement of consecutive units, identifies rotational axes, and calculates the corresponding Tait–Bryan angles. GeomeTRe’s parameters can enhance STRP annotation and classification by identifying variations in geometric arrangements among different functional groups. The package is fast and suitable for processing large protein structure datasets when repeat region information (e.g. from RepeatsDB) is available.

**Availability and implementation:**

GeomeTRe is available as a Python package; source code and documentation can be found at https://github.com/BioComputingUP/GeomeTRe.

## 1 Introduction

Tandem repeat proteins (TRPs) are prevalent throughout the tree of life and perform a broad range of functions (Marcotte *et al.* 1999, Kajava and Tosatto 2018). Structured tandem repeat proteins (STRPs) are a specific group characterized by conserved structural motifs in repetitive regions that do not necessarily share sequence similarity (Monzon *et al.* 2023). According to Kajava’s classification, STRPs can be grouped into five classes based on their shape and length (Kajava 2012). STRPs have distinctive structural and functional features, making them biologically significant subjects of study (Monzon *et al.* 2023, Arrías *et al.* 2024, Mac Donagh *et al.* 2024).

A deep understanding of repeat proteins’ structural characteristics is important for uncovering biological insights and for applications in *de novo* protein design (Vrancken *et al.* 2020). In protein design, STRPs—especially alpha-helical repeats—are frequently used as starting scaffolds due to their relatively simple structure (Brunette *et al.* 2015, Doyle *et al.* 2015). Studies in alpha-helical protein design suggest that STRPs can serve as scaffolds for biomaterials in nanotechnology and biomedical applications (Parmeggiani *et al.* 2015). For example, Park and colleagues controlled the curvature of repeat units to achieve a desired protein design (Park *et al.* 2015). In protein engineering, STRPs are valuable tools for biomedical applications due to their simplified sequence-structure relationships (Parmeggiani and Huang 2017).

The RepeatsDB classification, which extends Kajava’s, subdivides solenoid STRPs into alpha, beta, and alpha–beta topologies (Clementel *et al.* 2025). Solenoids can be described as a continuous superhelix, where the superhelix axis defines its “curvature” (Kobe and Kajava 2000). In this context, curvature results from the rotation of consecutive repeat units relative to each other, and is not necessarily aligned with the helical axis. Curvature can vary from large to small and serves as an additional parameter for STRP classification (Kobe and Kajava 2000). A recent review summarizes the classification of various alpha-solenoid subfolds based on curvature (Arrías *et al.* 2024). Twist is another geometrical property, defined as the coiling angle between consecutive units (Kobe and Kajava 2000). Twist can be right-handed (clockwise) or left-handed (anticlockwise). Most solenoid STRPs have an energetic preference for right-handed twists (Kobe and Kajava 2000), while others are left-handed (e.g. the LRR protein LegL7) (Batkhishig *et al.* 2021). For solenoids, properties such as curvature, twist, and handedness have mostly been described using a superhelical model. However, existing methods do not fully cover the structural diversity of STRPs. In Table 1, we report a comparison of tools designed to capture symmetries inside protein structures, highlighting the input, the output descriptors and the scope of structures they are designed for. Tools like SymD detect internal symmetry by aligning numerous fragments and applying circular permutations to find high-scoring matches, but they do not explicitly provide curvature, handedness, or twist values (Kim *et al.* 2010). Similarly, CE-Symm predicts internal symmetry in elongated and closed-repeat structures but cannot directly measure curvature or twist (Bliven *et al.* 2019). Another software, Ananas, calculates symmetry types in protein structures determining rotations and translations of symmetric subunits that best align them with minimal RMSD (Pagès and Grudinin 2018, Pagès *et al.* 2018).

**Table 1.** Overview of symmetry/repeat detection tools.

Tool	Input	Output	Scope
GeomeTRe	Structure^a^ + repeat units	Tait–Bryan angles along repeat units	Repeat structure
HELFIT	Structure^b^ + helical region	Helix parameters	Helical structure
RepeatParam	Structure^a^ + repeat units	Helix and superhelix parameters	Helical repeat structure
Rosetta^c^	Structure^a^	Helix parameters	Helical repeat structure
CE-Symm	Structure^a^	Symmetry type + region	Any structure
SymD	Structure^a^	Symmetry score	Any structure
Ananas	Structure^a^	Symmetry type	Any structure

a PDB file.

b Flat file with Cα coordinates.

c The output is generated by the “RepeatParameter” module of ROSETTA.

A different approach, HELFIT, uses a total least squares method to fit helical structures and defines features such as the helix axis, radius, pitch, handedness, and regularity (Enkhbayar *et al.* 2008).

Building on HELFIT, the recently proposed RepeatParam algorithm uses a helix-on-helix model to capture both the global architecture and the local superhelical structure of repeats (Pretorius and Murray 2024). While effective for helical repeats, these approaches may not perform well on non-helical repeat structures.

Originally developed for helical repeat protein design the “RepeatParameter” module of the ROSETTA software suite calculates angles for symmetry, but it is limited to helix structures, and not suitable for large-scale analysis (Fleishman *et al.* 2011).

Given these limitations, we developed GeomeTRe to characterize the geometrical properties of both open and closed-repeat proteins. The algorithm employs a circular fitting approach that provides a more flexible and accurate framework. The software is open-source and distributed as a Python package.

## 2 Materials and methods

GeomeTRe calculates geometrical properties of TRPs. It requires a protein structure and the start and end positions of each repeat unit (with optional insertion positions) as input. If insertion positions are provided, those segments are excluded to improve accuracy. For most known STRPs, repeat unit and insertion coordinates are available from the manually curated RepeatsDB database (Clementel *et al.* 2025) (Table S1, available as supplementary data at *Bioinformatics* online). The algorithm computes the three Tait–Bryan angles—yaw, pitch, and roll (Humphreys 1912, Allgeuer and Behnke 2015)—by simulating an airplane traversing the protein from its N-terminus to C-terminus. In this analogy, the airplane points to the centroid of the next repeat unit, and the angles correspond to the maneuvers required to move from one unit to the next. The strategy for defining the rotation axes used to calculate these angles is described below. The algorithm also determines handedness, defined by the roll direction of movement (clockwise/right-handed or anticlockwise/left-handed), and the sign of the pitch (positive for upward, negative for downward movement).

**Figure 1.**
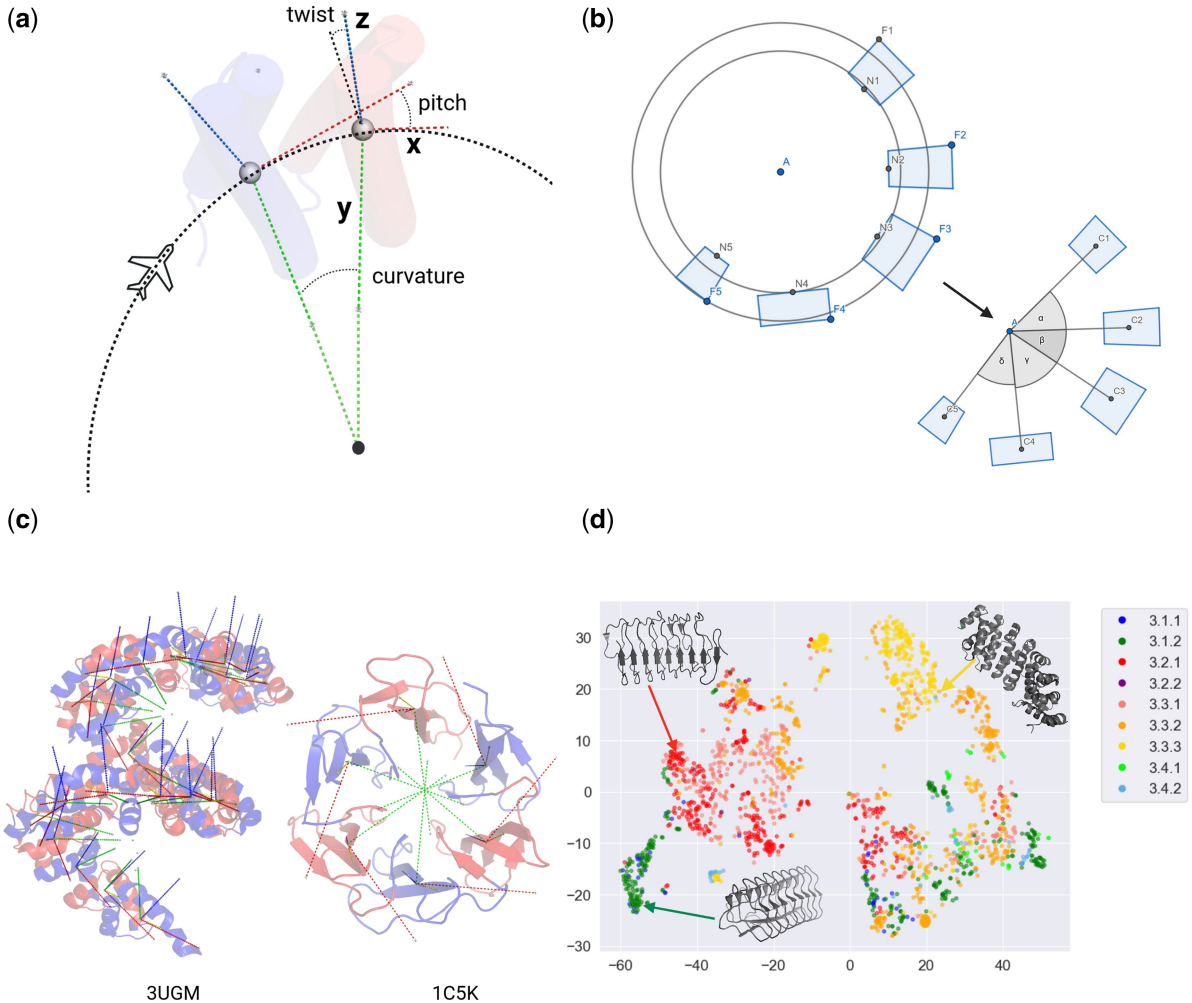
Definitions and distribution of GeomeTRe parameters in elongated STRPs. (A) The algorithm employs a circle-fitting method (panel B) through the geocenters of the repeat units to determine rotational axes. The twist axis (*x*, red) is tangential to the fitted circle, the pitch axis (*y*, green) points towards the circle’s center, and the curvature axis (*z*, blue) is perpendicular to the other two axes. Angles are computed by aligning two consecutive units after these axes are established. (B) The circle that best fits the unit coordinates is identified using a “widest crown” approach. All Cα atoms’ coordinates are projected onto the defined plane, and the widest crown between the innermost and outermost projected points, with reference to the initial circle for positioning (in/out), is determined. (C) Examples of GeomeTRe output on high curvature structures, illustrated using the GeomeTRe “draw” module. (D) t-SNE clustering of class 3 elongated STRPs based on GeomeTRe output parameters (mean and standard deviation of curvature, twist, and pitch). Colors correspond to fold classifications in RepeatsDB (3.1.1—right-handed beta-solenoid; 3.1.2—left-handed beta-solenoid; 3.2.1—high curvature alpha/beta-solenoid; 3.2.2—low curvature alpha/beta-solenoid; 3.3.1—low curvature alpha-solenoid; 3.3.2—high curvature alpha-solenoid; 3.3.3—corkscrew alpha-solenoid; 3.4.1—multimeric beta-hairpin; 3.4.2—monomeric beta-hairpin).

Curvature and twist have historically been used to describe STRP structures (Kobe and Kajava 2000). In our airplane analogy, these correspond to yaw and roll; however, an unambiguous description of the motion also requires calculating the pitch angle. This analogy between aircraft motion and the relative orientation of protein repeat units is illustrated in Fig. 1A.

Identifying and labeling rotation axes in proteins is not straightforward. Natural protein structures are irregular, and repeat units can lie on shapes for which it is difficult to define a dominant axis. Given the high degeneracy and structural variability among units in natural repeats, we divided the problem and made certain simplifying assumptions.

### 2.1 Curvature (yaw)

In the GeomeTRe algorithm, the roll axis (the direction of motion) corresponds to the vector connecting the centroids of two consecutive units. To identify the other two axes, the algorithm defines a reference plane by calculating the first two principal components of the coordinates of the centroids (geocenters) of the units within a window of six units.

The next step involves identifying a circle that best fits the units’ coordinates, which reflects the structure’s curvature. The algorithm employs a “widest crown” approach, as illustrated in Fig. 1B. Initially, it projects the centroids of the considered units onto the plane and fits an initial circle. Subsequently, it projects all the coordinates of the Cα atoms into the defined plane and identifies the widest crown between the innermost and outermost projected points, using the initial circle to determine their positions (in/out).

Curvature is defined as the angle at the circle’s center subtended by the centroids of two consecutive units and is computed for each unit. The use of a sliding window of six units, along with the “widest crown” approach, ensures stable and reliable results across various repeats, encompassing both elongated and closed structures. The choice of six units for the sliding window is based on empirical testing.

### 2.2 Twist (roll), pitch, and handedness

Twist and pitch angles are obtained by structurally aligning each repeat unit to its predecessor using TM-align (Zhang and Skolnick 2005). To ensure accuracy, we first rotate both units to a reference orientation defined by their pitch and twist axes. For each unit, the pitch axis is defined as the vector from the center of the fitted circle (see Section 2.1) to the unit’s centroid (i.e. the circle’s radius). The twist axis is computed by orthogonalizing the vector connecting two consecutive centroids with respect to the pitch axis, yielding the tangent to the circle at that unit. Prior to alignment, we rotate each unit by the cross-product of its twist and pitch axes to standardize their orientation.

After alignment, the resulting rotation is decomposed into twist (roll), pitch, and curvature (yaw) components. We discard the yaw component from this alignment, since curvature is already determined by the circle-fitting method. Twist handedness is defined as positive for clockwise rotations (negative for anticlockwise), and pitch handedness is positive when a unit moves upward (negative for downward).

### 2.3 Implementation

GeomeTRe is available as a Python package and can be used as a command-line tool or as a library. It relies on standard Python libraries (Numpy, Pandas, SciPy, scikit-learn, scikit-image, BioPython) and the *tmtools* package (which wraps TM-align). GeomeTRe also provides an option to visualize the rotational axes by launching a PyMOL session.

The program takes a protein structure and the start–end positions of its repeat units (and any insertions) as input. Repeat annotations for most known STRPs can be retrieved from RepeatsDB. The output is the set of geometrical parameters for each unit, returned as a Pandas DataFrame (which can be printed to standard output or saved to a CSV file).

GeomeTRe can also run in batch mode to process multiple PDB files in parallel. The algorithm is fast, taking less than a second per structure.

The GeomeTRe twist parameter shows a strong correlation with the twist parameter calculated by ROSETTA (Table S2, available as supplementary data at *Bioinformatics* online) with the exception of highly curved structures (e.g. 5cwq) for which ROSETTA overestimates the curvature. Additionally, the GeomeTRe curvature correlates with RepeatParam “twist” (theta), which represents the curvature of the main helix (Pearson’s correlation 0.38). Also, GeomeTRe twist correlates with RepeatParam “rise” (Pearson’s correlation 0.28). However, RepeatParam, in its current implementation, generates inaccurate curvature values for most of the nearly linear structures. The other tools listed in Table 1 provide parameters that are not comparable with those calculated by GeomeTRe.

## 3 Results and discussion

The GeomeTRe algorithm accurately determines the curvature of elongated and closed repeats, as well as challenging cases like linear structures with very low curvature. We analysed the distribution of GeomeTRe parameters (Figs S1–S5, available as supplementary data at *Bioinformatics* online) for high quality manually curated STRPs in RepeatsDB (8872 structures, 14 981 regions). Extended structures (class 3) vary in their number of repeat units, whereas closed STRPs (class 4) are constrained by their shape. This is reflected in the variability of geometrical parameters, especially curvature. For example, closed topologies like TIM barrels (class 4.1) and propellers (class 4.4) always have curvature above 0.5 radians, whereas elongated repeats like beta-solenoids (class 3.1) are rod-like with limited flexibility and exhibit very low curvature (Fig. S1, available as supplementary data at *Bioinformatics* online). For instance, the ice-binding protein (PDB 6eio), which helps protect bacteria from freezing (Mangiagalli *et al.* 2018), has a beta-solenoid structure with minimal curvature. Clathrin, a triskelion-shaped cytoplasmic protein (PDB 1b89) forms a polyhedral lattice to form protein-coated vesicles (Ybe *et al.* 1999), it is an alpha-solenoid and another example of a linear structure. In Fig. 1C, we show the output of the “draw” module of GeomeTRe for two high curvature structures, the TAL effector alpha-solenoid, which is a pathogenic bacterial protein and the TolB beta-propeller which mediates colicin (Carr *et al.* 2000, Mak *et al.* 2012).

For most topologies, pitch, twist, and curvature are positively correlated (Figs S6–S8, available as supplementary data at *Bioinformatics* online). The notable exception is the trefoil topology (class 4.3), where curvature is inversely correlated with both pitch and twist (Figs S7 and S8, available as supplementary data at *Bioinformatics* online). Overall, GeomeTRe parameters exhibit only weak inter-correlations, suggesting they capture distinct aspects of STRP architecture. To explore this, we performed t-SNE clustering on class 3 elongated repeats using these parameters (Fig. 1D). The resulting clusters align not only with the broad topology classes but also with specific fold subtypes.

We also observed that the mean of each parameter is positively correlated with its standard deviation across all topologies, except for class 4.3 (Figs S9–S11). The outlier behavior of topology 4.3 can be explained by the presence of two distinct fold populations in this class, which differ substantially in how their repeat units are organized.

### 3.1 Natural versus designed STRPs

In a “perfect repeat”, the rotations between consecutive units are nearly identical for all pairs. In natural proteins, however, these rotations can vary significantly depending on the degree of structural variability between units.

We compared the geometrical parameters of “natural” STRPs versus designed repeat proteins by extracting 76 designed repeat structures (132 repeat regions) from our dataset by checking the description available in the PDB, and compared their parameter distributions (Figs S12 and S13, available as supplementary data at *Bioinformatics* online).

The distribution of twist and curvature angles differs in these two sets. Natural STRPs display a broad range of curvature and twist, with values spanning [0, 2] radians, whereas designed repeats are limited to [0, 1] radians (Fig. S12, available as supplementary data at *Bioinformatics* online). In natural STRPs, moderate parameter correlations are observed for twist/pitch and pitch/curvature. In contrast, designed STRPs show consistently high correlations among all angle parameters (Fig. S13, available as supplementary data at *Bioinformatics* online). These pronounced correlations in designed proteins may indicate that structural constraints were optimized during design for specific functional or stability requirements.

## 4 Conclusions

We have presented GeomeTRe, a Python package for fast and accurate calculation of the main geometrical properties of TRPs using their structures and repeat annotations from RepeatsDB. The package calculates curvature, twist, pitch, and handedness, providing valuable insights for STRP classification. Our circular fitting method outperforms a superhelical fitting approach, particularly for β-solenoids and closed repeats, which have low curvature and a more planar organization.

The geometric parameters computed by GeomeTRe provide a quantitative description of repeat protein structures, enabling more refined structural classifications and potential refinement of the current RepeatsDB classification. Furthermore, the descriptors provided by GeomeTRe can act as valuable input features for machine learning-driven methodologies, such as protein design pipelines, and can support manual curation of unit definitions within RepeatsDB by offering real-time feedback on parameter changes based on unit definitions.

## Supplementary Material

btaf395_Supplementary_Data

## Data Availability

GeomeTRe is available in GitHub at https://github.com/BioComputingUP/GeomeTRe. All input data and results from this study are available at https://github.com/BioComputingUP/GeomeTRe_results.
