# Supplementary material for: GeomeTRe: accurate calculation of geometrical descriptors of tandem repeat proteins
Source: Bioinformatics. 2025 Jul 10;41(7):btaf395. doi: 10.1093/bioinformatics/btaf395 (PMC12308183; doi:10.1093/bioinformatics/btaf395)
Supplement: btaf395_Supplementary_Data [file btaf395_supplementary_data.pdf]

Supplementary data

GeomeTRe: accurate calculation of geometrical descriptors of tandem repeat proteins

Zarifa Osmanli[1,^], Elisa Ferrero[1,2,^], Alexander Miguel Monzon[1], Silvio C E Tosatto[1,2], Damiano Piovesan[1,*]

[1]Department of Biomedical Sciences, University of Padova, Padova 35121, Italy.
[2]Galileian School of Higher Education, University of Padova, Padova 35132, Italy.
[3]Institute of Biomembranes, Bioenergetics and Molecular Biotechnologies, National Research Council (CNR-IBIOM), Bari, Italy.

[^]These authors contributed equally.

*Corresponded author: damiano.piovesan@unipd.it

Table of contents

Table of contents
1. RepeatsDB classification
2. Comparison with ROSETTA
3. Parameters distributions
4. Parameters correlations
5. Mean and standard deviation (std) correlations
6. Comparison between natural and designed STRPs

1. RepeatsDB classification

Table S1. Classification of elongated and closed structured tandem repeat proteins (STRPs) in RepeatsDB (repeatsdb.org).

ID	Name
3.1	Beta-solenoid
3.2	Alpha/beta solenoid
3.3	Alpha-solenoid
3.4	Beta hairpins
4.1	TIM-barrel
4.2	Beta-barrel/beta hairpins
4.3	Trefoil
4.4	Propeller
4.5	Alpha/beta prism
4.6	Alpha-barrel
4.7	Alpha/beta barrel
4.8	Aligned prism

2. Comparison with ROSETTA

Table 1. Comparison of GeomeTRe and a Rosetta twist calculation on designed helical repeats.

ID	PDB ID	ROSETTA	GeomeTRe
DHR4	5cwb	0.58	0.55
DHR5	5cwc	0.06	0.09
DHR7	5cwd	0.66	0.60
DHR8	5cwf	0.40	0.36
DHR10	5cwg	0.03	0.08
DHR14	5cwh	0.17	0.01
DHR18	5cwi	0.36	0.32
DHR49	5cwj	0.18	0.04
DHR53	5cwk	0.29	0.44
DHR54	5cwl	0.26	0.22
DHR64	5cwm	0.37	0.09
DHHR71	5cwn	0.23	0.08
DHR76	5cwo	0.27	0.23
DHR79	5cwp	0.42	0.26
DHR81	5cwq	1.00	0.08

Comparison of GeomeTRe and Rosetta estimates for twist on experimentally validated designed repeat structures. Angle values are reported in radians. For those entries that are not in RepeatsDB the unit definition has been performed manually. All structures contain four units.

3. Parameters distributions

Topology 4.7 is excluded in Figure S1-S3 due to the small number of representative STRPs available in RepeatsDB.

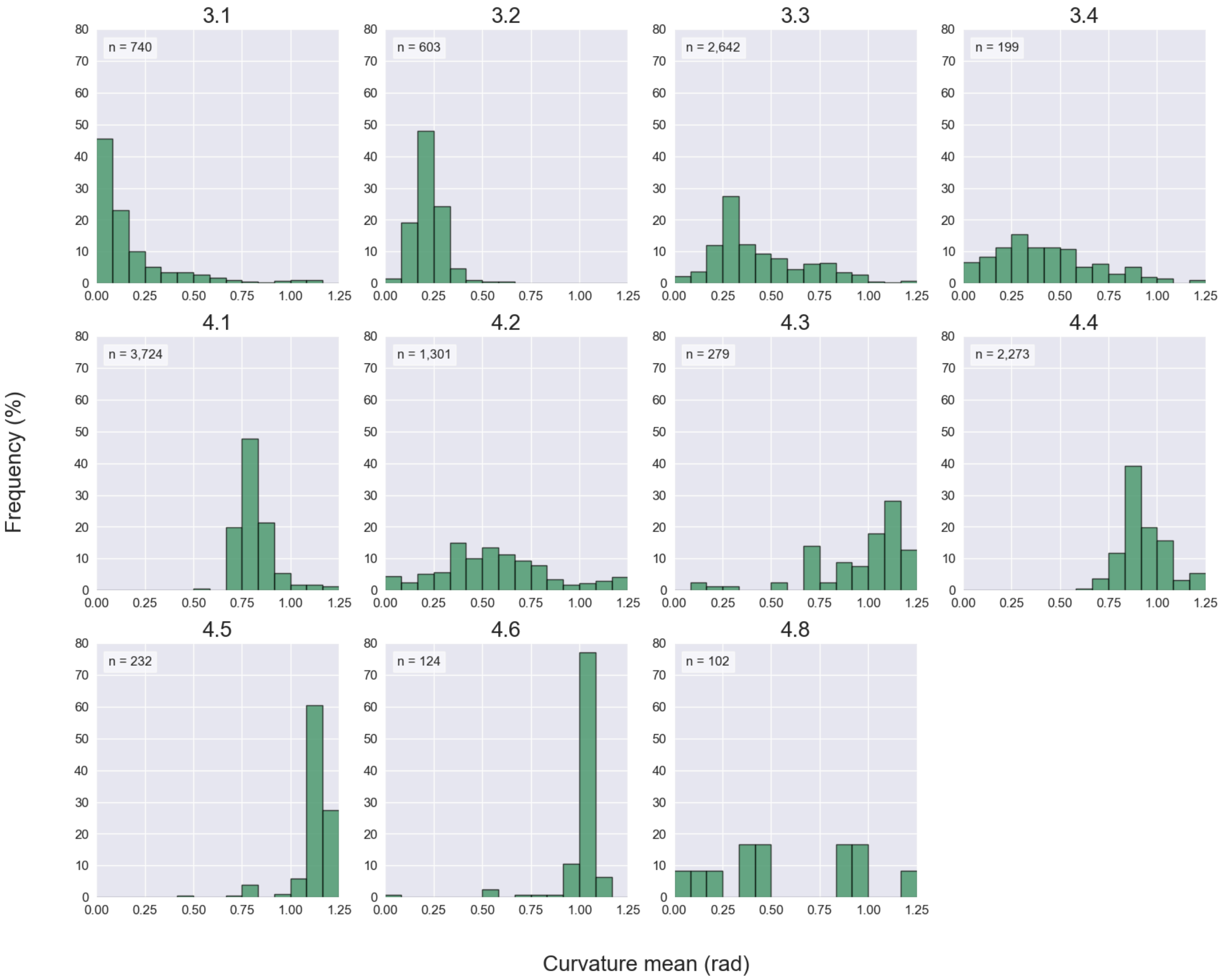

Figure S1. Frequency distribution of mean of curvature parameter across topologies of elongated (class 3) and closed (class 4) STRPs.

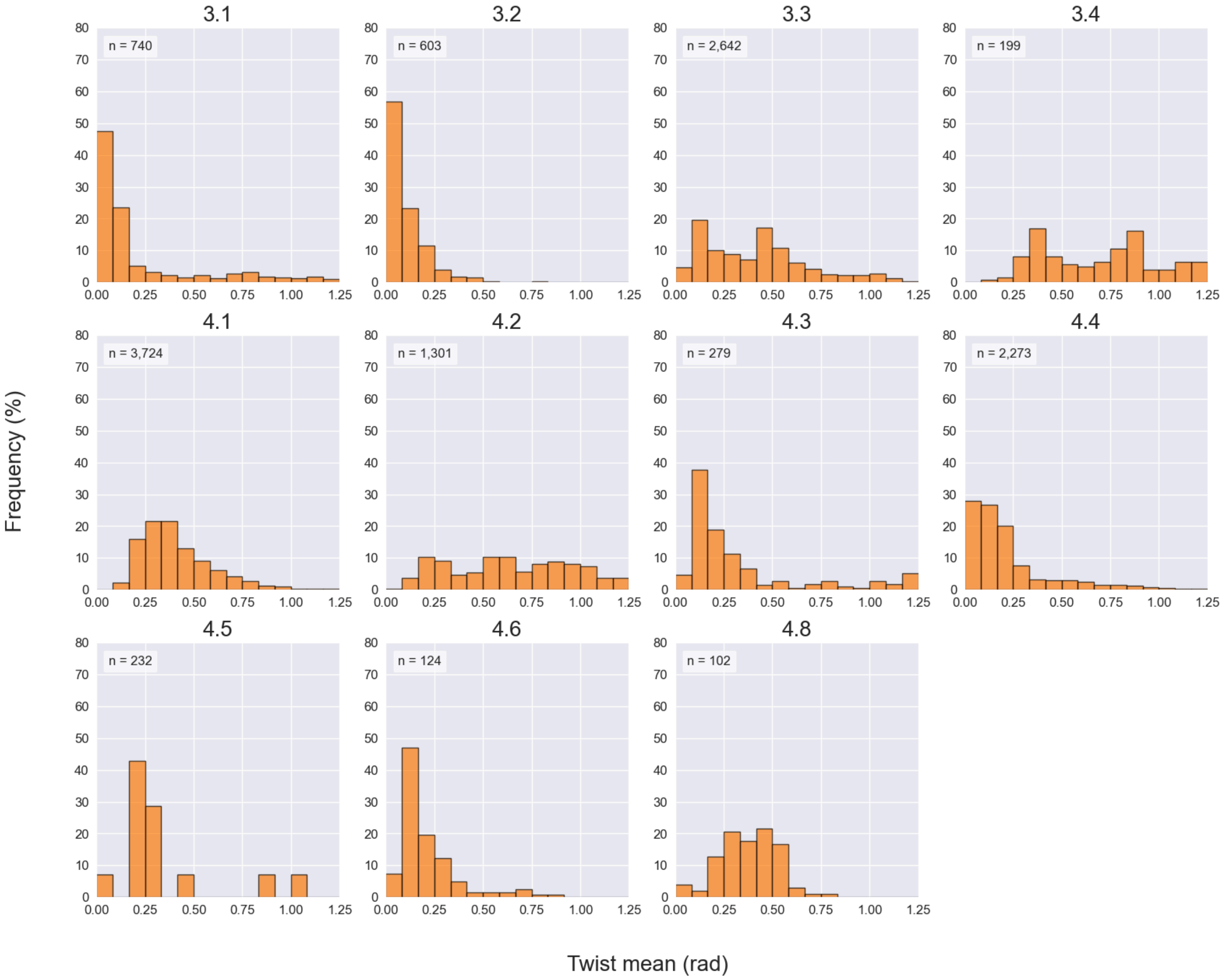

Figure S2. Frequency distribution of mean of twist parameter across topologies of elongated (class 3) and closed (class 4) STRPs.

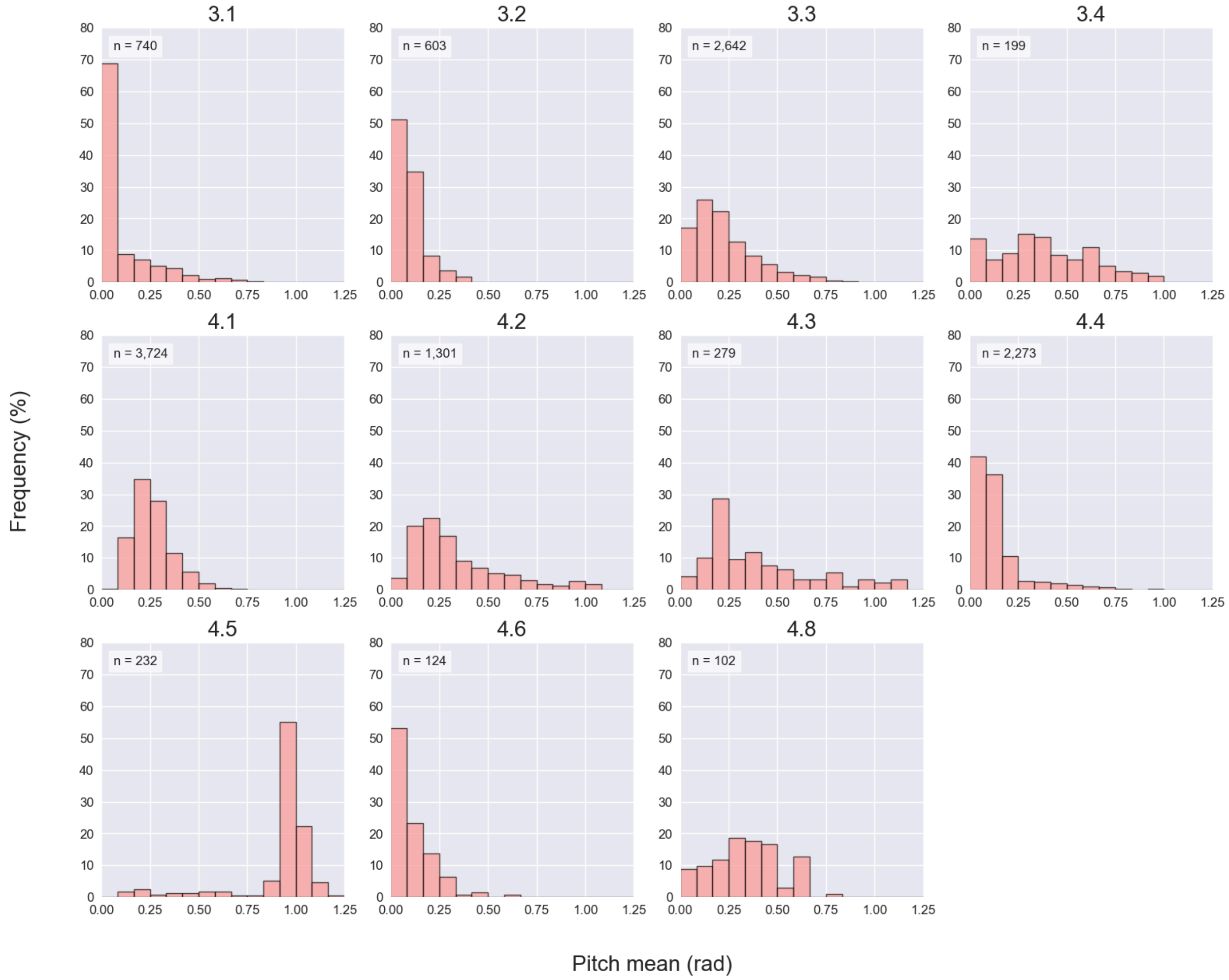

Figure S3. Frequency distribution of mean of pitch parameter across topologies of elongated (class 3) and closed (class 4) STRPs.

The handedness of the protein structure can be either right handed (+1) or left handed (-1). For some STRPs the handedness changes direction along the region and resulting average values are continuous. For the handedness statistics in order to exclude structures which are too linear or that change direction, we considered only STRP regions with both an average higher than 0.1 and a standard deviation lower than 0.1. Structures with less than 6 units are excluded.

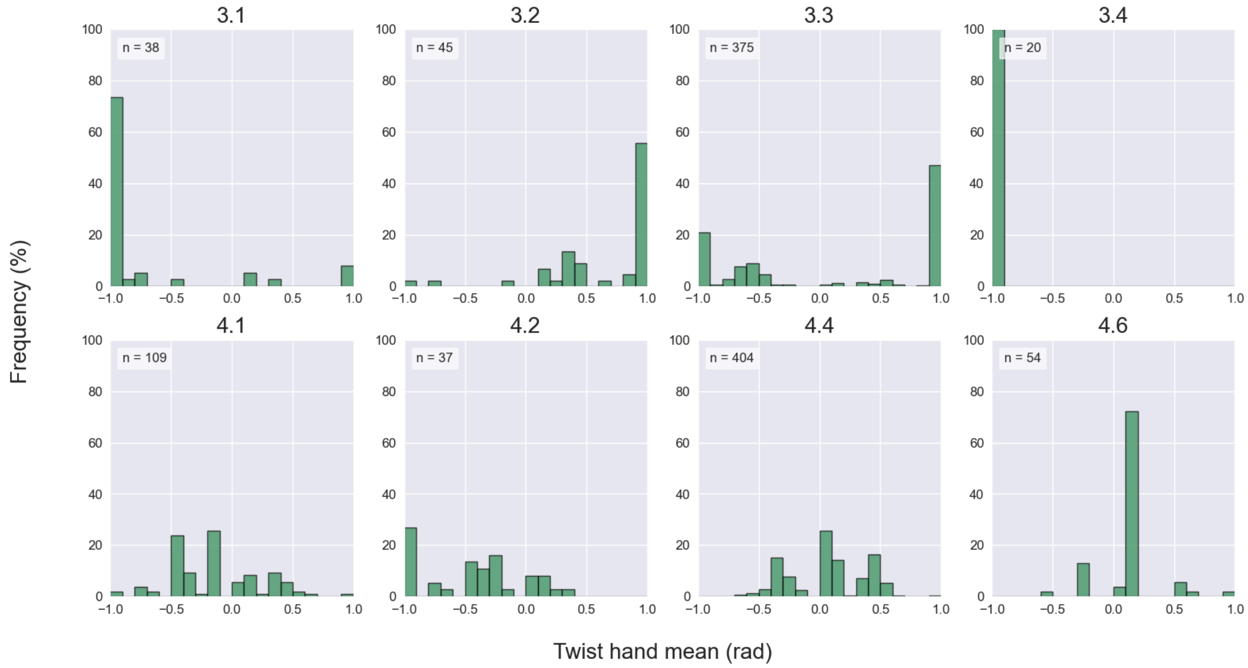

Figure S4. Frequency distribution of mean twist handedness among topologies of elongated (class 3) and closed (class 4) STRPs. Less than 10 structures in topologies 4.3, 4.5, 4.7 and 4.8 passed the filtering criteria and therefore are not shown.

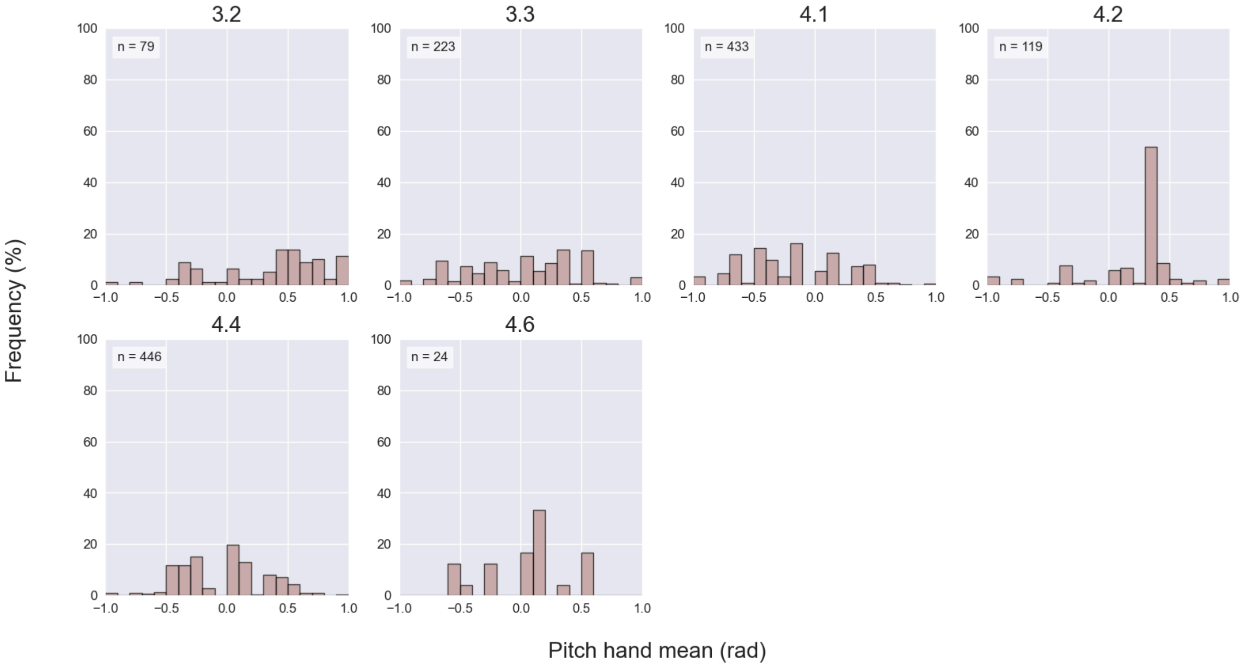

Figure S5. Frequency distribution of mean pitch handedness among topologies of elongated (class 3) and closed (class 4) STRPs. Less than 10 structures in topologies 3.1, 3.4, 4.3, 4.5, 4.7 and 4.8 passed the filtering criteria and therefore are not shown.

4. Parameters correlations

Topology 4.7 is excluded due to the small number of available STRPs in RepeatsDB. The number of repeat regions considered in each topology (subplot) is indicated with *n*.

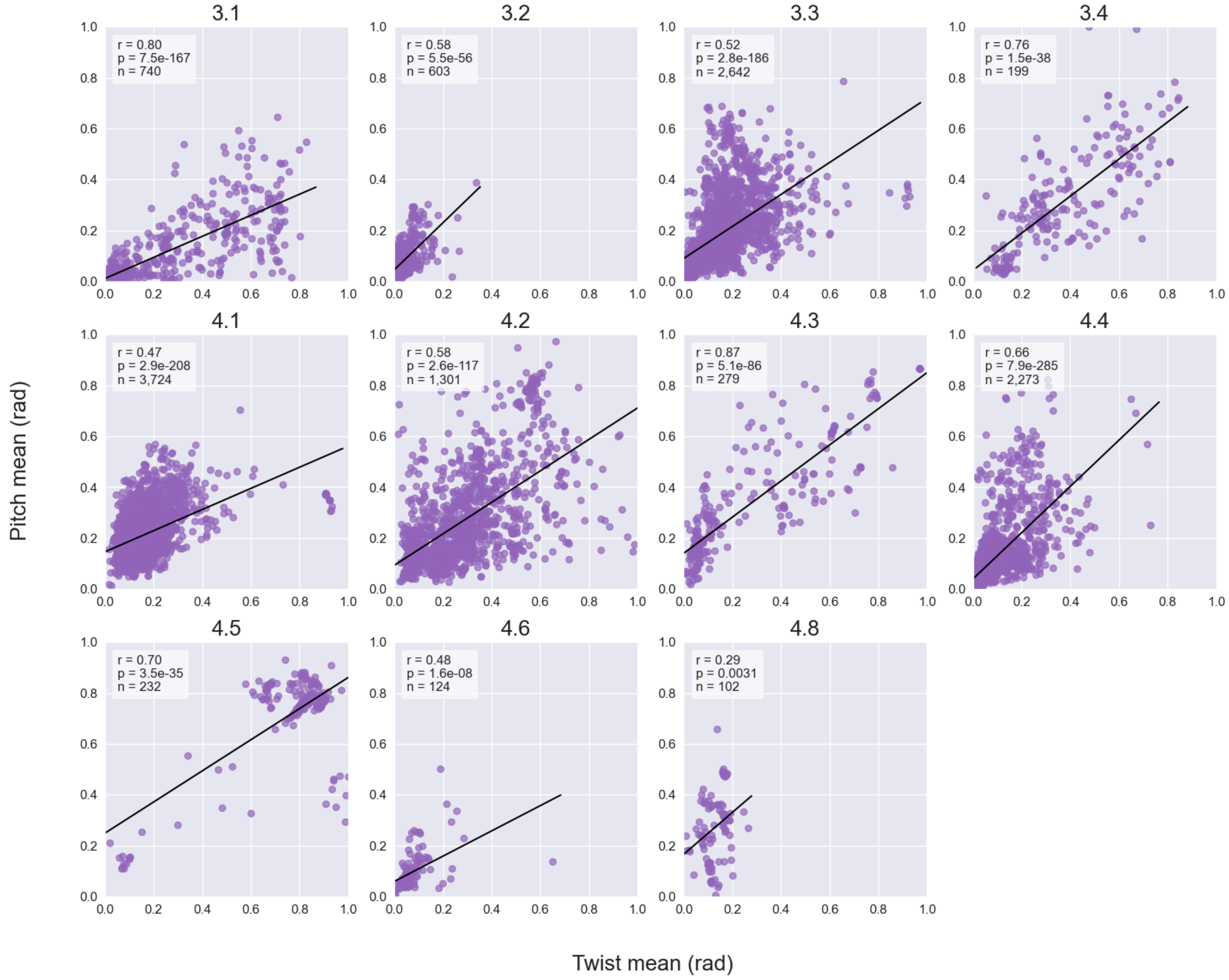

Figure S6. Correlation of mean of pitch and twist parameters for elongated (class 3) and closed (class 4) STRPs at the topology level.

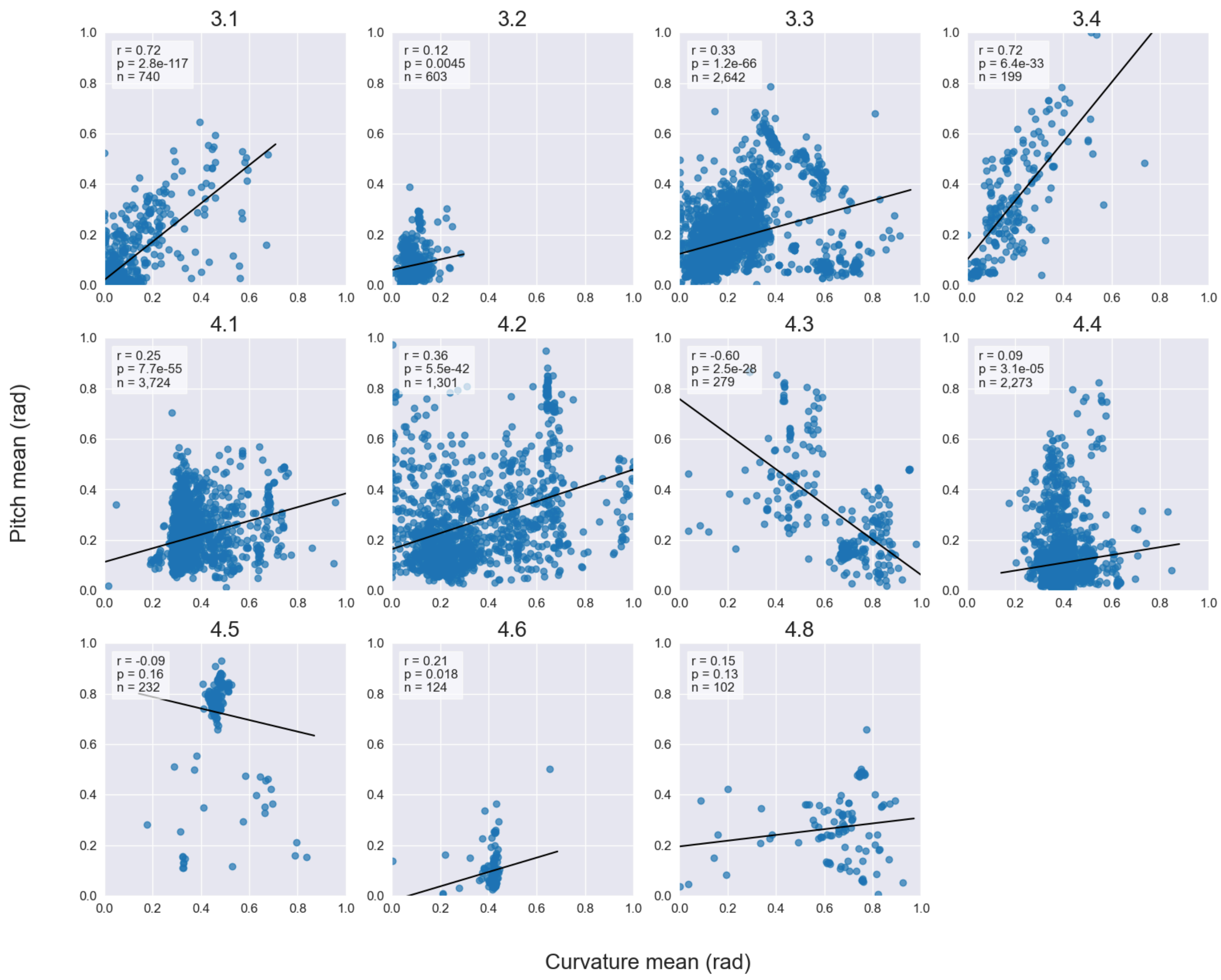

Figure S7. Correlation of mean of curvature and pitch parameters for elongated (class 3) and closed (class 4) STRPs at the topology level.

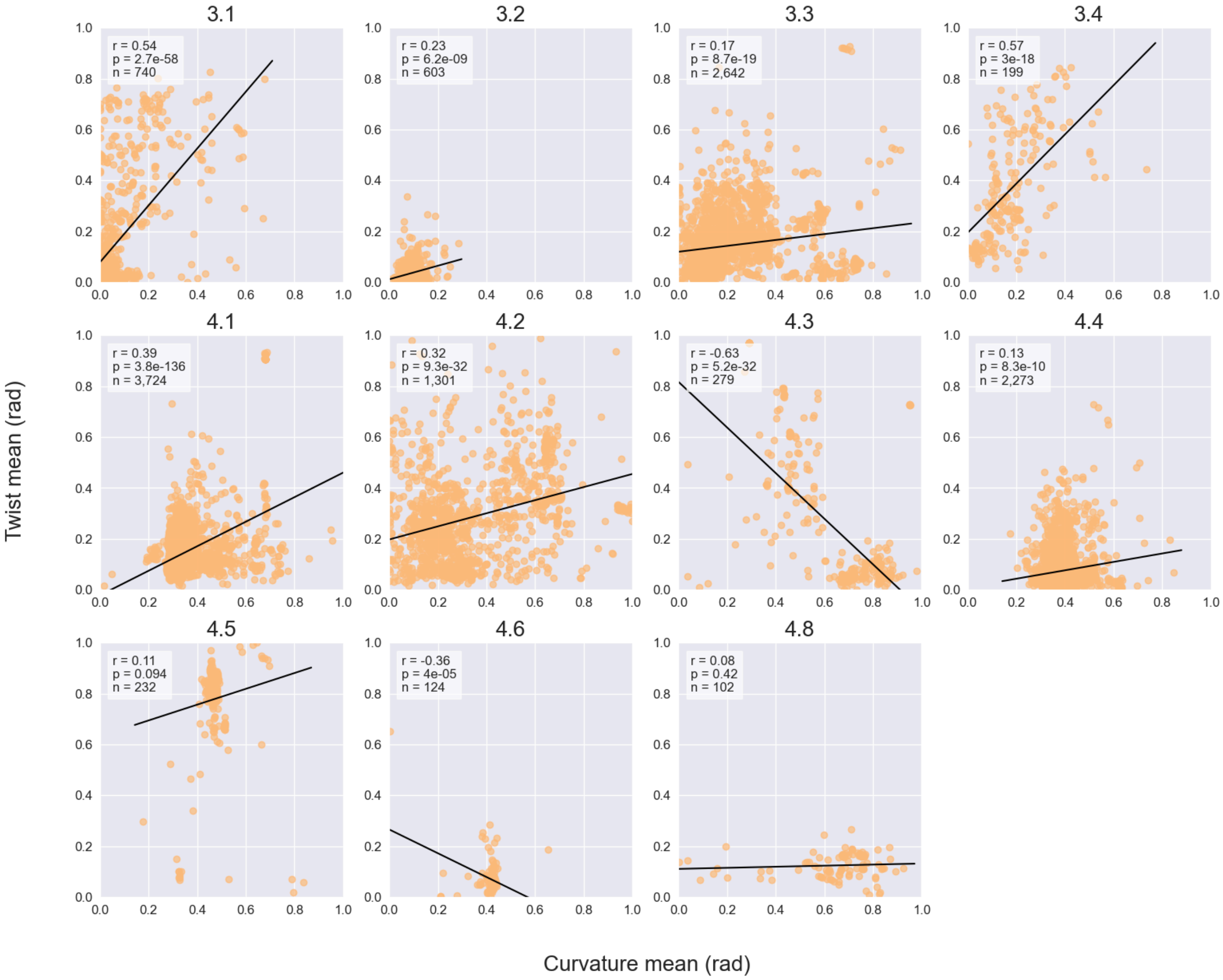

Figure S8. Correlation of mean of curvature and twist parameters for elongated (class 3) and closed (class 4) STRPs at the topology level.

5. Mean and standard deviation (std) correlations

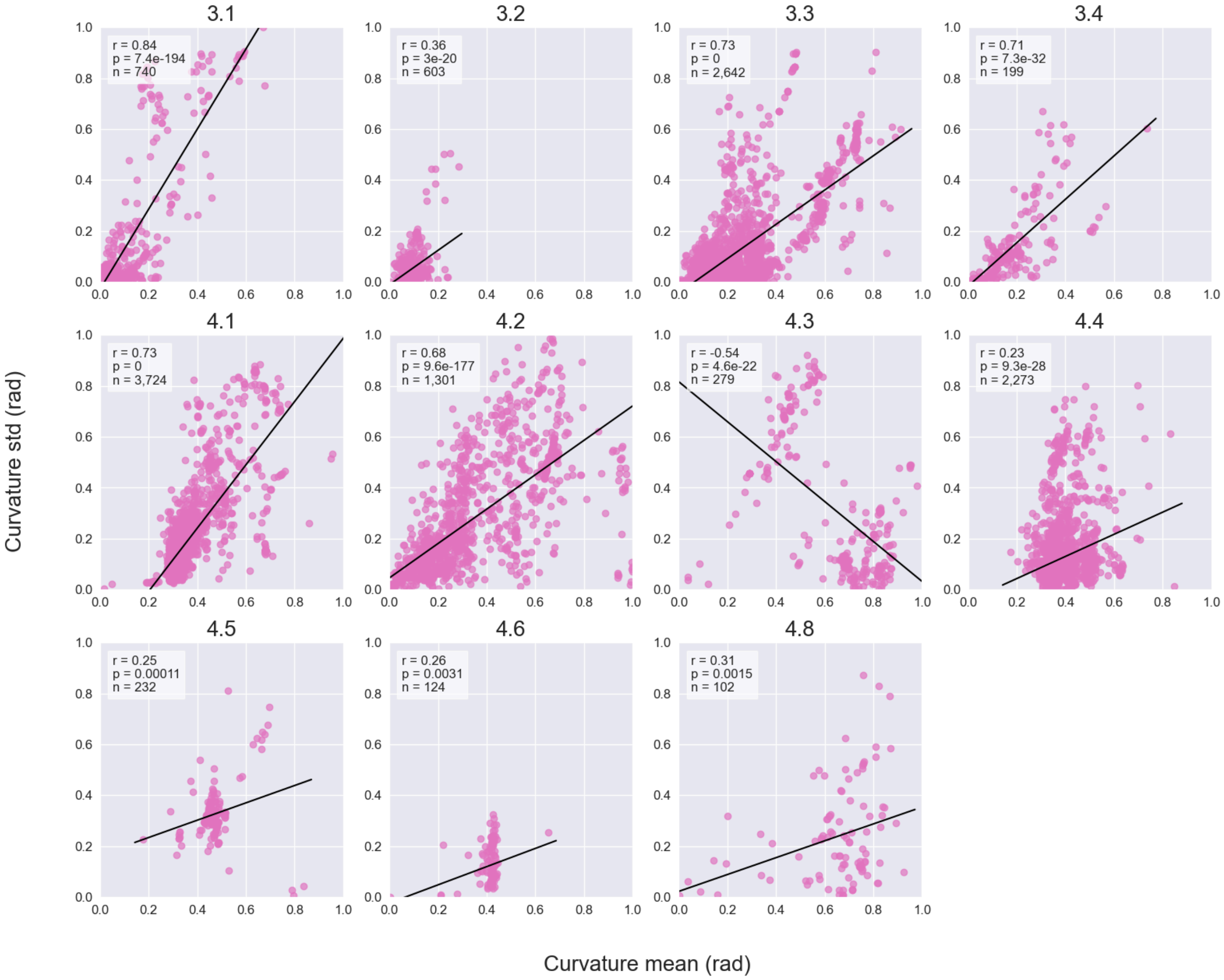

Figure S9. Correlation distribution of mean and standard deviation of curvature across topologies of elongated (class 3) and closed (class 4) STRPs.

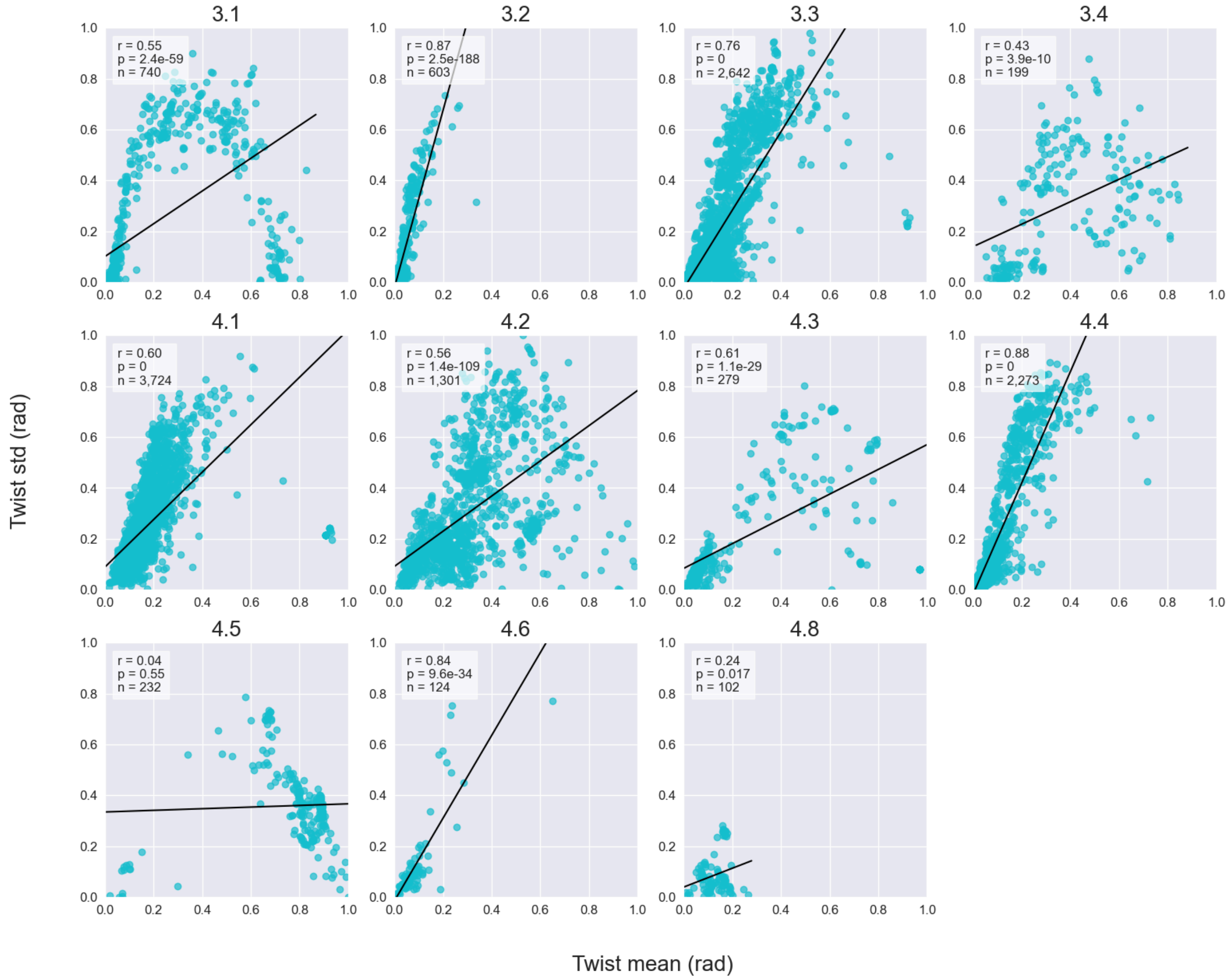

Figure S10. Correlation distribution of mean and standard deviation of twist across topologies of elongated (class 3) and closed (class 4) STRPs.

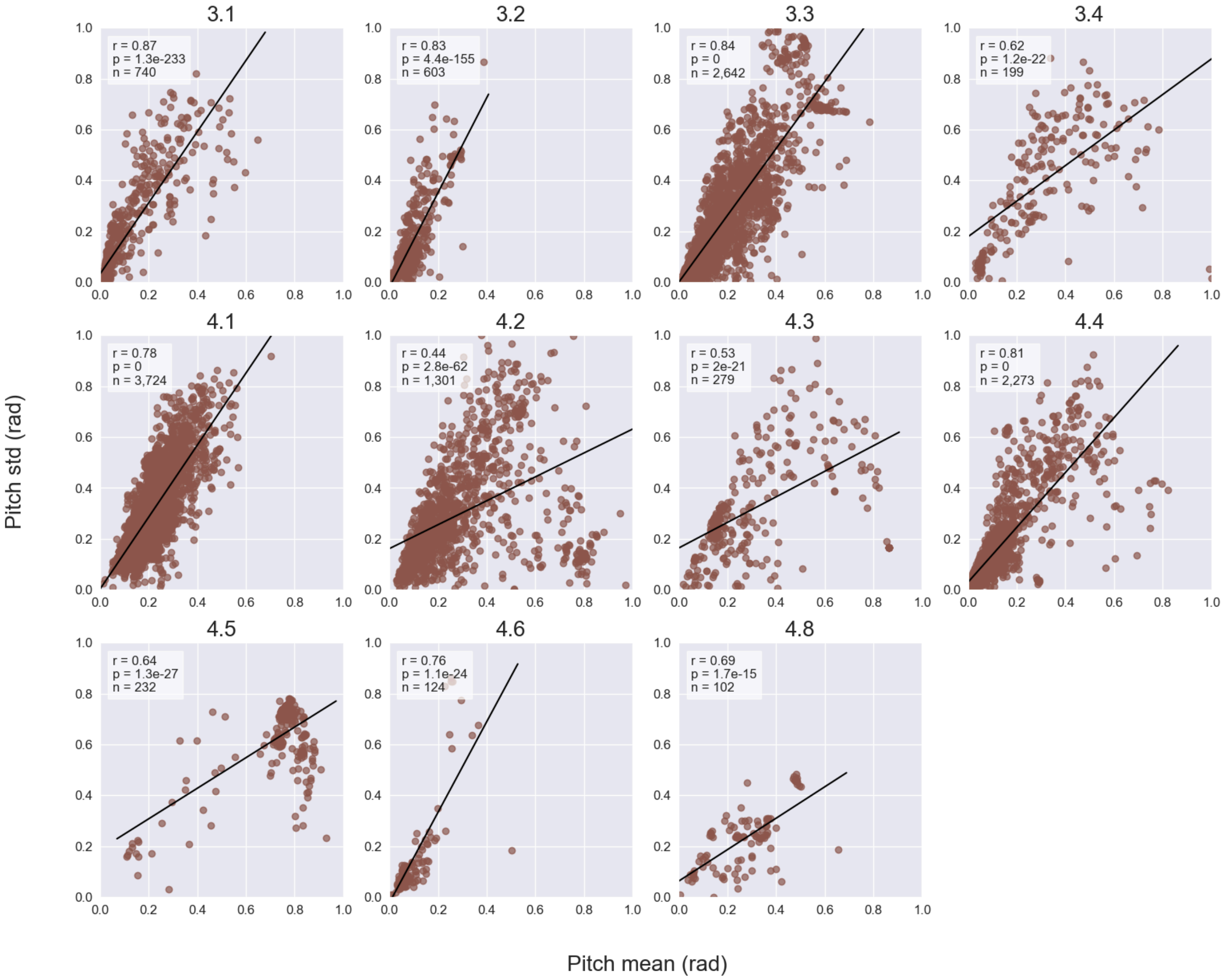

Figure S11. Correlation distribution of mean and standard deviation of pitch across topologies of elongated (class 3) and closed (class 4) STRPs.

6. Comparison between natural and designed STRPs

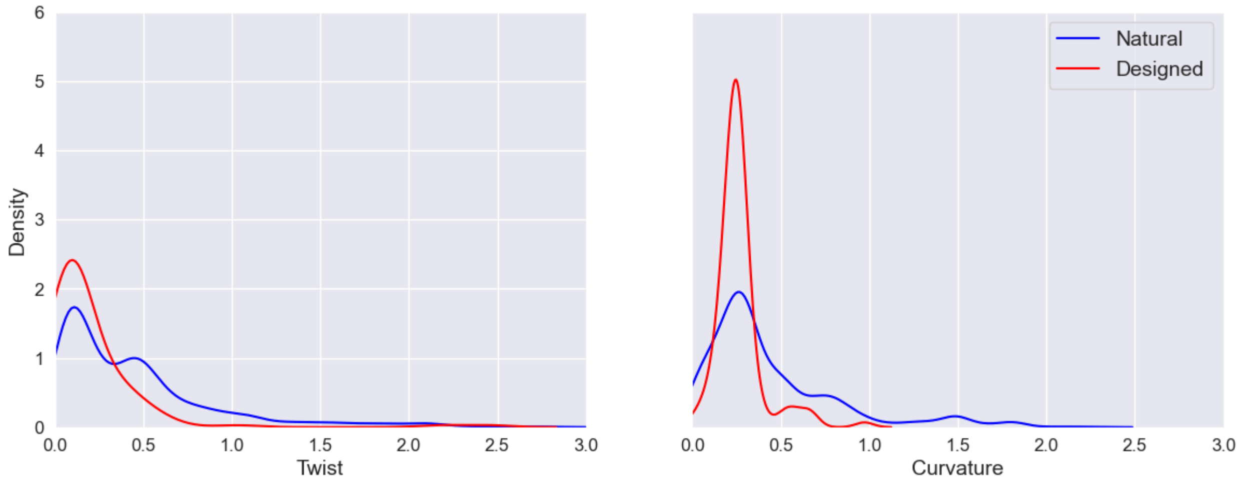

Figure S12. Kernel Density Estimate (KDE) distribution of twist and curvature parameters for 14,849 natural and 132 designed STRPs regions of class 3 and class 4.

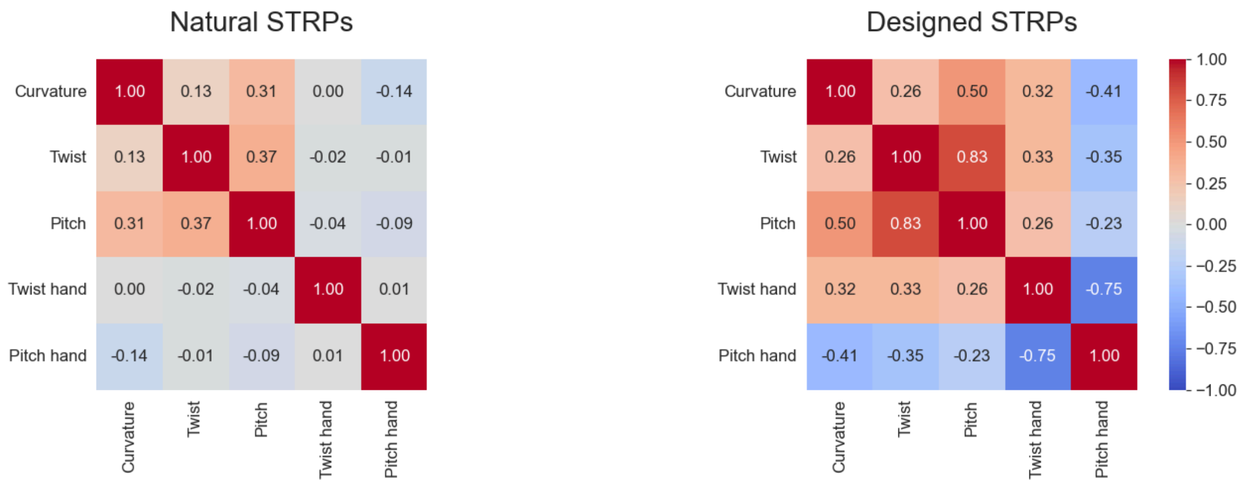

Figure S13. Heatmap of Pearson Correlation of geometry parameters (curvature, twist, pitch, twist handedness, pitch handedness) in 14,849 natural and 132 designed STRPs regions.
